# Supplementary material for: Targeting ADAR stability: a novel approach to combat glioblastoma
Source: Front Oncol. 2026 Aug 6;16:1827793. doi: 10.3389/fonc.2026.1827793 (PMC13489870; doi:10.3389/fonc.2026.1827793)
Supplement: Supplementary file 1 [file SupplementaryFile1.zip › Supplementary file 1/Fatty_acid_elongation.pdf]

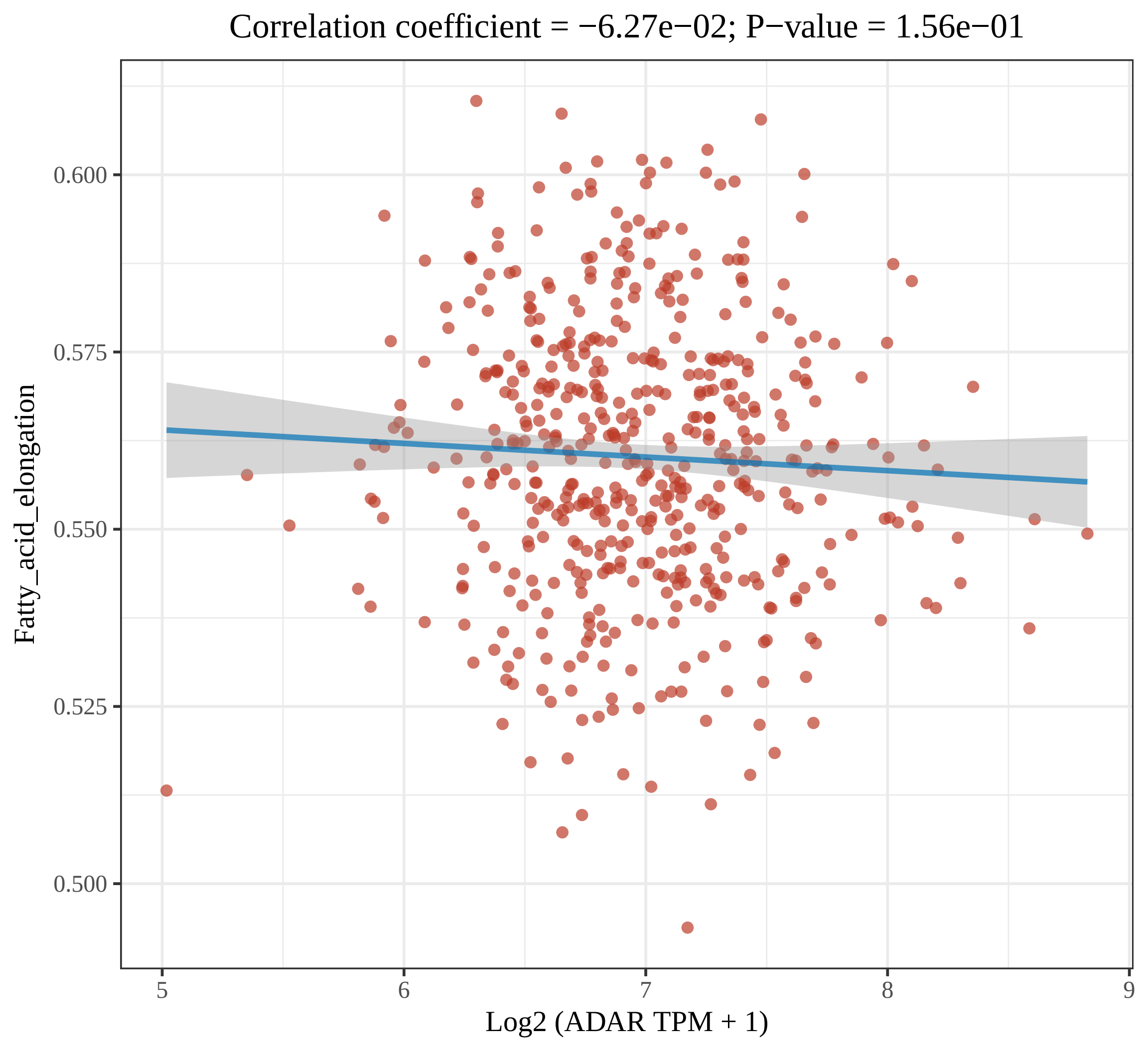

Correlation coefficient = −6.27e−02; P−value = 1.56e−01
Fatty_acid_elongation
0.600
0.575
0.550
0.525
0.500
5
6
7
8
9
Log2 (ADAR TPM + 1)
